# N-Terminal Arginines Modulate Plasma-Membrane Localization of Kv7.1/KCNE1 Channel Complexes

**DOI:** 10.1371/journal.pone.0026967

**Published:** 2011-11-04

**Authors:** Zenawit Girmatsion, Peter Biliczki, Ina Takac, Christin Schwerthelm, Stefan H. Hohnloser, Joachim R. Ehrlich

**Affiliations:** 1 Division of Cardiology, Section of Electrophysiology, J.W. Goethe-University, Frankfurt, Germany; 2 Institute of Cardiovascular Physiology, J.W. Goethe-University, Frankfurt, Germany; University of Frankfurt - University Hospital Frankfurt, Germany

## Abstract

**Background and Objective:**

The slow delayed rectifier current (I_Ks_) is important for cardiac action potential termination. The underlying channel is composed of Kv7.1 α-subunits and KCNE1 β-subunits. While most evidence suggests a role of KCNE1 transmembrane domain and C-terminus for the interaction, the N-terminal KCNE1 polymorphism 38G is associated with reduced I_Ks_ and atrial fibrillation (a human arrhythmia). Structure-function relationship of the KCNE1 N-terminus for I_Ks_ modulation is poorly understood and was subject of this study.

**Methods:**

We studied N-terminal KCNE1 constructs disrupting structurally important positively charged amino-acids (arginines) at positions 32, 33, 36 as well as KCNE1 constructs that modify position 38 including an N-terminal truncation mutation. Experimental procedures included molecular cloning, patch-clamp recording, protein biochemistry, real-time-PCR and confocal microscopy.

**Results:**

All KCNE1 constructs physically interacted with Kv7.1. I_Ks_ resulting from co-expression of Kv7.1 with non-atrial fibrillation ‘38S’ was greater than with any other construct. Ionic currents resulting from co-transfection of a KCNE1 mutant with arginine substitutions (‘38G-3xA’) were comparable to currents evoked from cells transfected with an N-terminally truncated KCNE1-construct (‘Δ1-38’). Western-blots from plasma-membrane preparations and confocal images consistently showed a greater amount of Kv7.1 protein at the plasma-membrane in cells co-transfected with the non-atrial fibrillation KCNE1-38S than with any other construct.

**Conclusions:**

The results of our study indicate that N-terminal arginines in positions 32, 33, 36 of KCNE1 are important for reconstitution of I_Ks_. Furthermore, our results hint towards a role of these N-terminal amino-acids in membrane representation of the delayed rectifier channel complex.

## Introduction

The slow delayed rectifier current (I_Ks_) is important for cardiac repolarization. One of the current’s key functions is to prevent excessive action potential prolongation during adrenergic stimulation. It represents an important constituent of the “repolarization reserve” [1]. The single-transmembrane segment β-subunit KCNE1 modulates the function of the six-transmembrane segment, pore-forming α-subunit Kv7.1 [2], [3]. Within the heart KCNE1 is the major interacting ß-subunit associating with Kv7.1. The interaction between these proteins determines I_Ks_ properties and modulates current characteristics (eliminating ionic current inactivation, increasing unitary conductance and slowing activation) [4], [5]. Most of the interactions underlying this modulation have been localized to the transmembrane domain and the C-terminus of KCNE1 [6]-[8]. Solid evidence suggests that the intracellular end of the KCNE1 transmembrane segment (C-terminus) comes into close proximity of the Kv7.1 S4–S5 linker and subsequently modulates ion channel gating [9]. The role of the KCNE1 N-terminus for delayed rectifier channel interaction and ionic current modulation remains largely unexplored.

**Figure 1:**
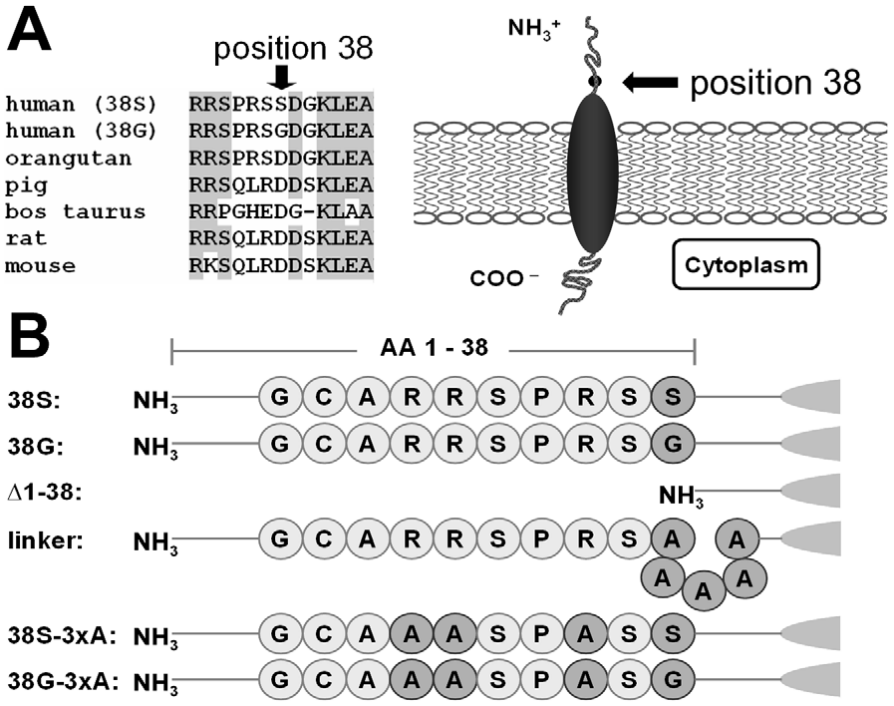
Schematic of KCNE1 and constructs. This figure schematically illustrates KCNE1 structure and mutants used in the present study. **A, left**, alignment of KCNE1 sequences from various mammalian species. Grey underlines conserved residues. Glycine at position 38 is not strongly conserved among species providing no first-glance evidence for evolutionary importance. **A, right**, schematic of KCNE1 at the membrane with the N-terminal part oriented towards the cell exterior and C-terminus towards the cytosol. **B**, schematic of KCNE1 N-terminal constructs and mutations created for the present study. Ten N-terminal amino-acids (AA) illustrate differences between KCNE1 constructs. Position 38 carries a glycine in the wild-type (common allele) and is associated with atrial fibrillation. Position 38 carries a serine in the prevalent single nucleotide polymorphism. One of the constructs contained an N-terminal truncation (‘Δ1-38’), another one (‘linker’) replaced position 38 by 5 alanines. Additionally, three positively-charged arginines at positions 32, 33 and 36 have been exchanged for alanines in order to probe the role of these AA in KCNE1 function.

An N-terminal single nucleotide polymorphism results in an amino-acid change (G38S) in an unconserved KCNE1 position and is highly prevalent (Fig. 1A). It can be found in up to ∼50% of individuals in different ethnicities [10], [11]. A population study described an association of the common allele KCNE1-38G with atrial fibrillation, a highly prevalent human arrhythmia. Odds ratios for atrial fibrillation occurrence were 2.16 with one ‘38G’ allele and 3.58 with two ‘38G’ alleles [10]. The atrial fibrillation-associated KCNE1-38G allele results in reduced I_Ks_ density possibly due to impaired membrane trafficking of I_Ks_ channels [12]. The underlying structure-function correlation of this N-terminal region has not yet been studied.

The present study examined the hypothesis that arginines in position 32, 33 and 36 within the KCNE1 N-terminus are specifically important for membrane representation of KCNE1/Kv7.1 channel complexes and for I_Ks_ modulation.

## Results

### Expression of KCNE1 mutants and interaction with Kv7.1

**Figure 2:**
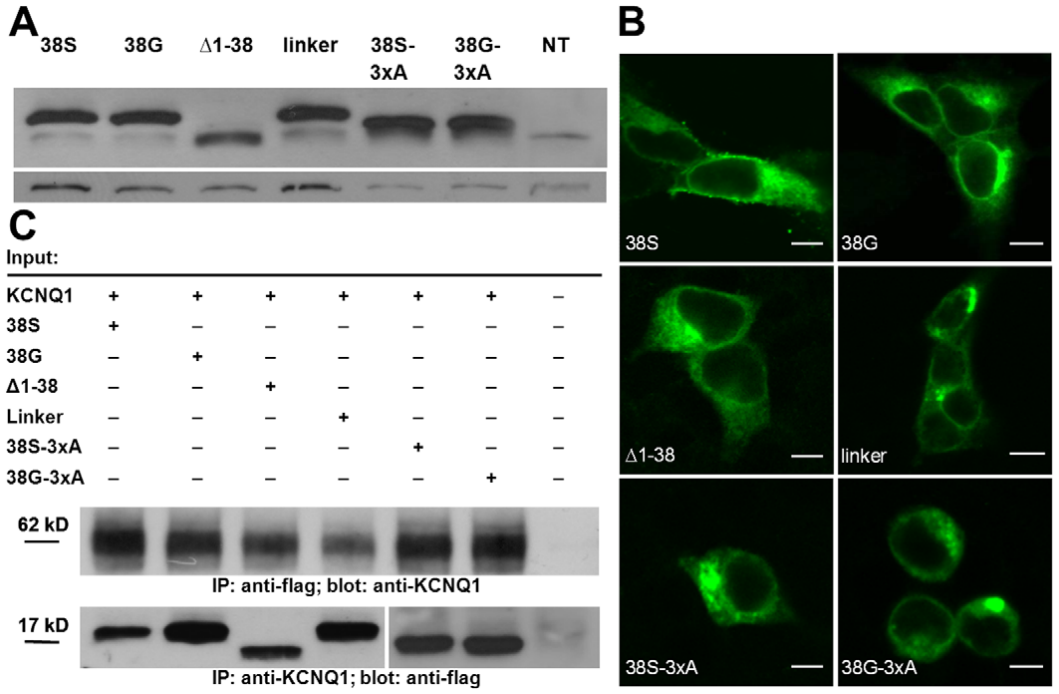
Immunodetection of heterologously expressed constructs. Immunodetection of flag-tagged KCNE1 constructs. **A**, crude membrane preparations from HEK cells transiently transfected with respective KCNE1 constructs. Actin (∼42 kD) is shown as loading control. **B**, effective co-immunoprecipitation (IP) occurred for Kv7.1 with all flag-tagged KCNE1 constructs. The upper blot shows protein samples from HEK cells precipitated by anti-flag and bands detected by anti-Kv7.1. The lower blot shows samples precipitated by anti-Kv7.1 and bands detected by anti-flag antibody (n = 2 experiments each). **C** shows respective confocal images of KCNE1 subunits expressed without Kv7.1. Bars represent 5 µm. Images are representative of at least 5 different experiments. NT – non-transfected control, IP – immunoprecipitation.

Analysis of KCNE1 protein expression by Western blotting revealed bands at the apparent molecular weight of ∼17 kD for both '38S' and '38G'. The molecular weight of ‘Δ1-38’ was slightly smaller due to the truncation and ‘linker’ was heavier due to four additional amino-acids. ‘38S-3xA’ and ‘38G-3xA’ appeared slightly smaller than ‘38S’ and ‘38G’. Fig. 2A shows crude membrane preparations from cells transfected with respective flag-tagged KCNE1 constructs illustrating similar overall protein expression. All constructs effectively co-immunoprecipitated with Kv7.1 indicating physical interaction between α- and β-subunits (Fig. 2B). Confocal microscopy illustrated no differences in subcellular localization of KCNE1 subunits expressed alone (Fig. 2C).

### Electrophysiological studies

**Figure 3:**
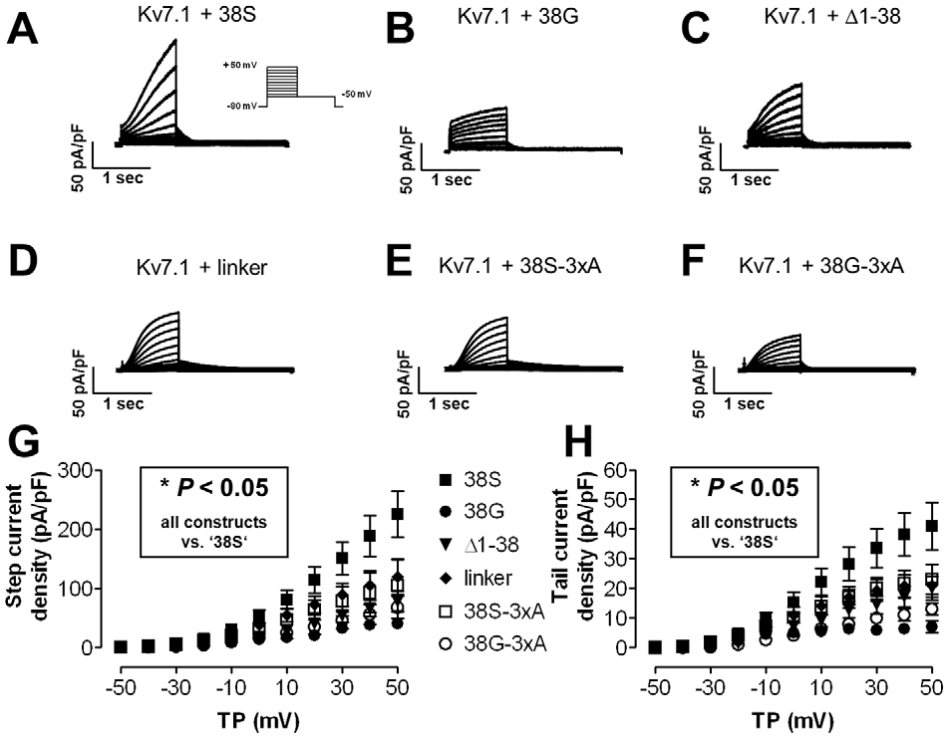
Electrophysiological properties. **A–F**, representative ionic currents elicited by whole-cell patch-clamp with the protocol shown in inset A. **G**, current-voltage relations of mean±SEM depolarization induced activating step-current densities from cells transfected with Kv7.1 plus various KCNE1 constructs. **H,** mean±SEM current-voltage relationships of repolarization induced tail-current densities. P-values are shown for currents recorded from cells transfected with Kv7.1+’38S’ vs. all other constructs. The remainder of constructs did not lead to significantly different current sizes compared with ‘38G’ (*P* = n.s.). TP – test potential.

Ionic currents were recorded from HEK and CHO cells to exclude cell-line specific effects, results were comparable (not shown). Figs. 3A–F illustrate representative currents from HEK cells expressing Kv7.1 with respective KCNE1 constructs. Currents were largest with Kv7.1 and ‘38S’ co-expression, while all other currents were within a similar order of magnitude. I_Ks_ densities obtained with depolarizing pulses to +50 mV were: ‘38S’: 225±39 pA/pF [n = 18], ‘38G’: 41±9 pA/pF [n = 9], ‘Δ1–38’: 78±17 pA/pF [n = 9], ‘linker’: 119±30 pA/pF [n = 5], ‘38S-3xA’: 105±43 pA/pF [n = 6], ‘38G-3xA’: 68±19 pA/pF [n = 6]; *P*<0.05 for all constructs vs. ‘38S’, comparison of ‘38G’ vs. the remaining constructs indicated no differences (*P* = non-significant [n.s.], Fig. 3G). Similarly, deactivating tail currents were greatest with KCNE1-38S (after depolarization to +50 mV: ‘38S’: 41±8 pA/pF, ‘38G’: 7±2 pA/pF, ‘Δ1-38’: 20±4 pA/pF, ‘linker’: 21±4 pA/pF, ‘38S-3xA’: 23±5 pA/pF, ‘38G-3xA’: 13±2 pA/pF; *P*<0.05 for all vs. ‘38S’, *P* = n.s. for ‘38G’ vs. remaining constructs; n of each group as detailed_above; Fig. 3H).

**Figure 4:**
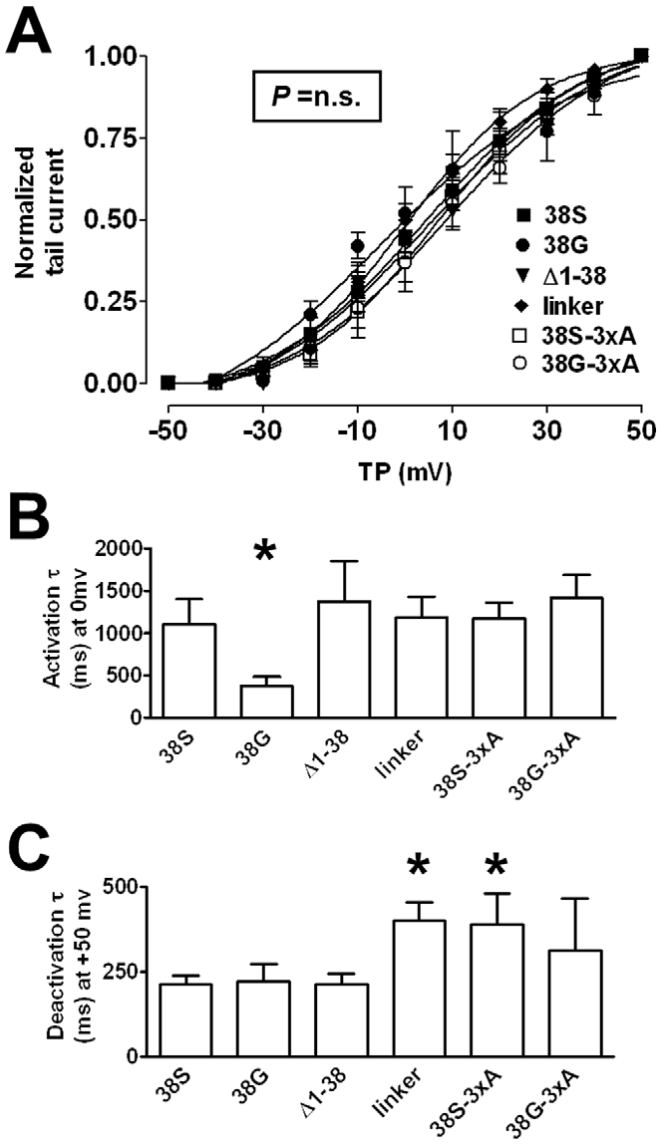
Biophysical characteristics. **A**, illustrates half-activation voltages (V_50_) of currents resulting from expression of Kv7.1 with respective constructs. Mean±SEM V_50_ values were similar between constructs ‘38S’: 5.8±3.7 mV, ‘38G’: −2.7±4.1 mV, ‘Δ1-38’: 6.0±4.1 mV, ‘linker’: 1.0±2.5 mV, ‘38S-3xA’: 3.4±8.6 mV, ‘38G-3xA’: 8.7±6.8 mV; *P* = n.s vs. ‘38S’. Lines shown are Boltzmann fits to mean data (obtained with the formula: A = A_0_/(1+exp[(V_50_-V)/S])). **B** shows results of mono-exponential fits (y = A^(−t/τ)^+C) to activating currents with time-constants plotted over a test potential of 0 mV. **C** shows results of mono-exponential fits to deactivating ionic currents. Currents obtained from co-transfection of Kv7.1 with ‘linker’ and ‘38S-3xA’ deactivated more slowly than currents obtained with the remainder of the constructs (*P*<0.05 vs. ‘38S’). TP – test potential.

Half-activation voltages (V_50_) obtained from Boltzmann fits to normalized tail currents did not differ between constructs: ‘38S’ (5.8±3.7 mV), ‘38G’ (-2.7±4.1 mV), ‘Δ1-38’ (6.0±4.1 mV), ‘linker’ (1.0±2.5 mV),’38S-3xA’ (3.4±8.6 mV) and ‘38G-3xA’ (8.7±6.8 mV); *P* = n.s. vs.’38S’ and *P* = n.s. for all remaining constructs vs. ‘38G’; n of each group as detailed above; (Fig. 4A). Slope-factors were unaffected (‘38S’: 14.3±0.8, ‘38G’: 15.0±5.0, ‘Δ1-38’: 18.4±2.8, ‘linker’: 15.2±1.3, ‘38S-3xA’: 14.8±1.3, ‘38G-3xA’: 15.3±1.1; *P* = n.s.). Activation kinetics obtained by fitting mono-exponential functions to activating ionic currents were similar between groups (time-constants at depolarization to 0 mV were: ‘38S’: 1103±297 ms, ‘Δ1-38’: 1372±479 ms, ‘linker’: 1185±245 ms, ‘38S-3xA’: 1175±187 ms and ‘38G-3xA’: 1413±276 ms (*P* = n.s.). Time-constants of the activation for ‘38G’: 373±111 ms were smaller (indicating faster activation) compared with ‘38S’: 1103±297 ms and the remainder of the constructs (*P*<0.05, Fig. 4B). The n of each group was as detailed above. Deactivation of currents evoked from cells after depolarization to +50mV, transfected with Kv7.1 and KCNE1 constructs ‘linker’ and ‘38S-3xA’ deactivated more slowly than currents from cells transfected with Kv7.1 and ‘38S’ (time-constants: ‘38S’: 213±24 ms, ‘38G’: 220±51 ms, ‘Δ1-38’: 212±30 ms, ‘linker’: 400±56 ms, ‘38S-3xA’: 390±89 ms, ‘38G-3xA’: 311±154 ms; *P*<0.05 for ‘linker’ and ‘38S-3xA’ vs. ‘38S’; *P* = n.s. for remaining constructs vs. ‘38S’; there were no differences among remaining constructs and ‘38G’; n of each group as detailed above; Fig. 4C).

**Figure 5:**
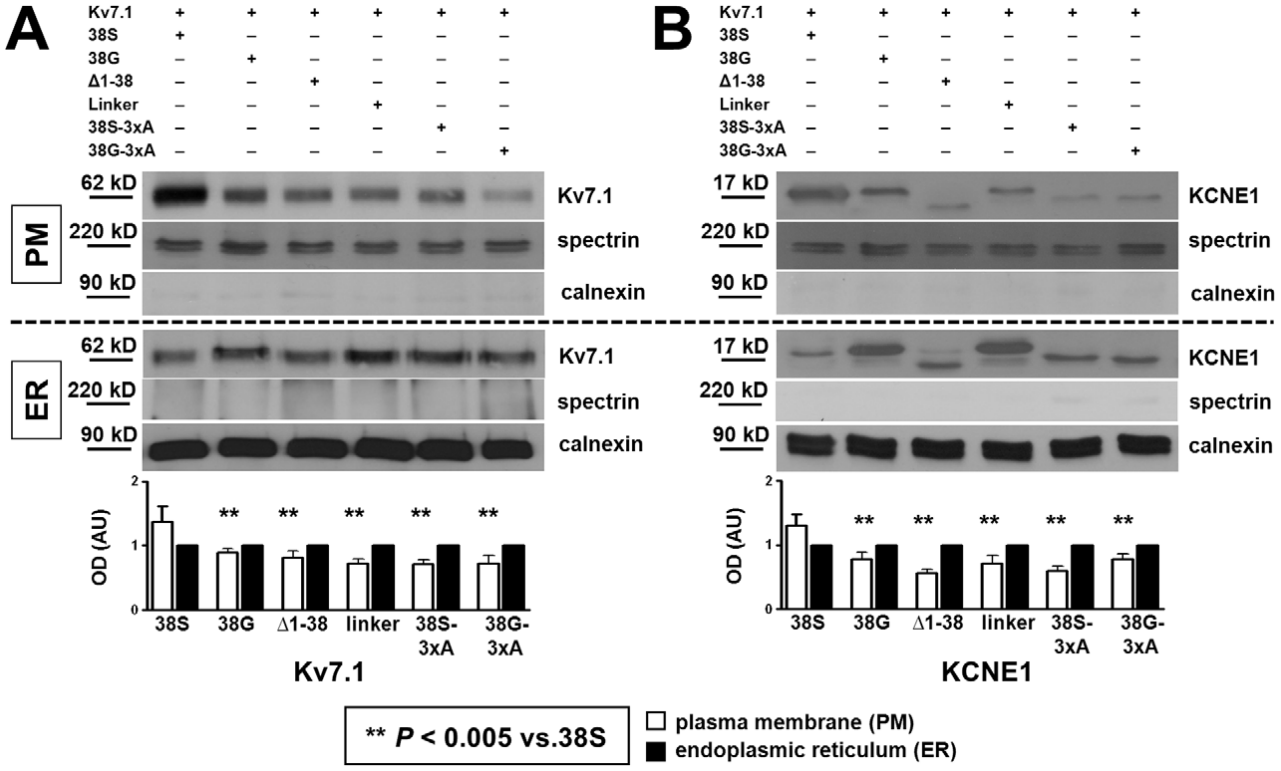
Membrane representation of Kv7.1 and KCNE1. This figure illustrates results from cell compartment fractionation experiments yielding plasma-membrane (PM) and endoplasmic reticulum (ER) fractions. **A, B** show representative blots of cells transfected with Kv7.1 and KCNE1 constructs. The upper part of each panel shows a blot of plasma-membrane proteins, the lower part of each panel illustrates the endoplasmic reticulum-fraction. Spectrin was used as loading control for plasma-membrane, calnexin for endoplasmic reticulum. Freedom from contamination of plasma-membrane and endoplasmic reticulum fractions is illustrated by representation of spectrin and calnexin, respectively. **A**, mean±SEM optical densities (OD) quantified from immunoblots of Kv7.1 illustrate an increase in Kv7.1 protein at the plasma-membrane and **B** shows a similarly greater amount of KCNE1-38S protein at the plasma-membrane. The respective bar graphs below represent the mean±SEM data. White bars represent Kv7.1 or KCNE1 protein content normalized to spectrin as loading control for plasma-membrane fractions, black bars represent protein content normalized to calnexin as loading control for endoplasmic reticulum fractions. Data of plasma-membrane protein content were thereafter normalized against endoplasmic reticulum (n = 4).

### Plasma-membrane localization

After having found increased ionic currents with Kv7.1 and ‘38S’ compared to Kv7.1 and all of the N-terminal constructs and no changes between currents recorded from any of the other constructs in comparison to ‘38G’, we evaluated the effect of KCNE1 constructs on Kv7.1 and KCNE1 plasma-membrane localization. Fig. 5 shows representative western blots from fractionation experiments of cells transfected with Kv7.1 and respective flag-tagged KCNE1 constructs. Freedom from contamination of plasma-membrane and endoplasmic reticulum fractions is illustrated by absence of spectrin (plasma-membrane marker) in endoplasmic fraction and absence of calnexin (endoplasmic reticulum marker) in membrane fractions. Kv7.1 (Fig. 5A) and KCNE1 (Fig. 5B) were immunodetected in respective fractions and spectrin and calnexin were further used as expression control. Blots illustrate greater Kv7.1 abundance at the plasma-membrane and less in the endoplasmic reticulum when cells were co-transfected with KCNE1-38S than the other constructs (mean±SEM data: ‘38S’: 1.3±0.2, ‘38G’: 0.9±0.1, ‘Δ1-38’: 0.8±0.1, ‘linker’: 0.7±0.1, ‘38S-3xA’: 0.7±0.1, ‘38G-3xA’: 0.7±0.1 arbitrary units [AU]; *P*<0.005 vs. ‘38S’ for all, Fig. 5A). Plasma-membrane distribution of respective KCNE1 constructs was modulated in analogy to Kv7.1 distribution (‘38S’: 1.4±0.1, ‘38G’: 0.8±0.1, ‘Δ1-38’: 0.6±0.1, ‘linker’: 0.7±0.1, ‘38S-3xA’: 0.6±0.1, ‘38G-3xA’: 0.8±0.1 AU; *P*<0.005 vs. ‘38S’ for all; Fig. 5B).

These data are consistent with electrophysiological measurements and suggest that the N-terminus and specifically the three arginines are important for plasma-membrane representation of the KCNE1 β-subunit as results obtained with alanine-scanning constructs were comparable to those obtained with the N-terminally truncated construct.

### Immunofluorescent studies

**Figure 6:**
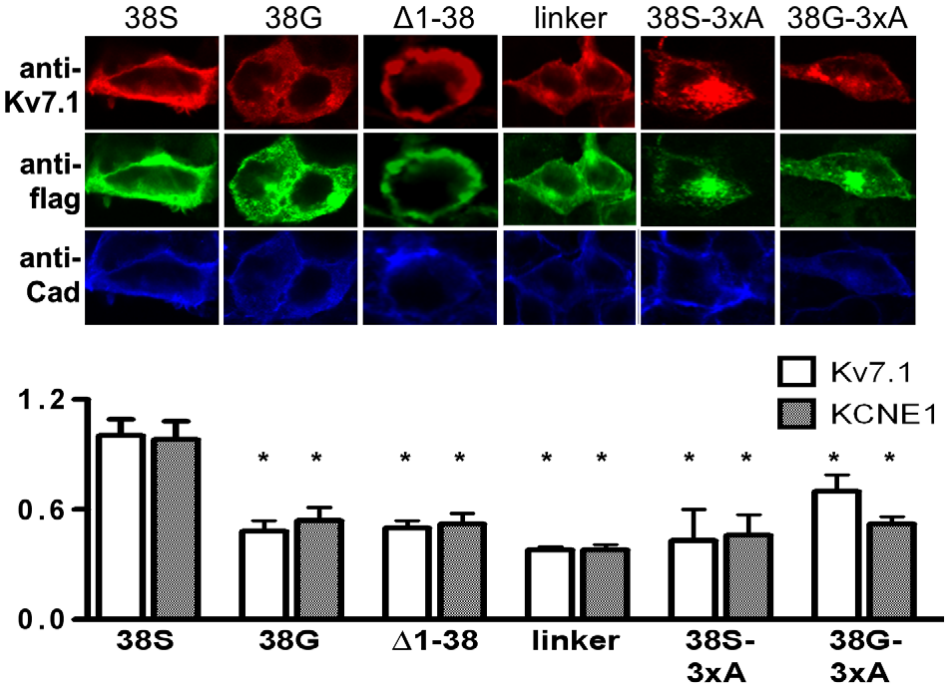
Immunofluorescent studies of membrane representation. Confocal images of HEK cells transfected with Kv7.1 and flag-tagged KCNE1 constructs. The top row localizes Kv7.1 (red) within cells. The middle row shows the distribution of KCNE1 (green), bottom panels illustrate the plasma-membrane marker pan-cadherin (blue). Bars represent 10 µm. The respective bar graphs below represent the mean±SEM data of Kv7.1 and KCNE1 correlation with plasma-membrane marker pan-cadherin. White bars represent Kv7.1/pan-cadherin ratio, hatched bars are KCNE1/pan-cadherin ratio. Results are from 4-9 experiments each. Cad – cadherin, other abbreviations as above.

In order to further substantiate these results we applied confocal microscopy (Fig. 6) to double-labelled cells co-transfected with Kv7.1 and respective flag-tagged KCNE1 constructs. Pan-cadherin staining was performed to mark plasma-membranes. All KCNE1 constructs co-localized with the α-subunit. Upon laser line-scanning with identical gain settings, Kv7.1 and KCNE1 membrane fluorescence appeared consistently greater in the presence of KCNE1-38S compared with the other KCNE1 constructs.

### mRNA transcription of Kv7.1 (α-) and KCNE1 (ß-subunits)

**Figure 7:**
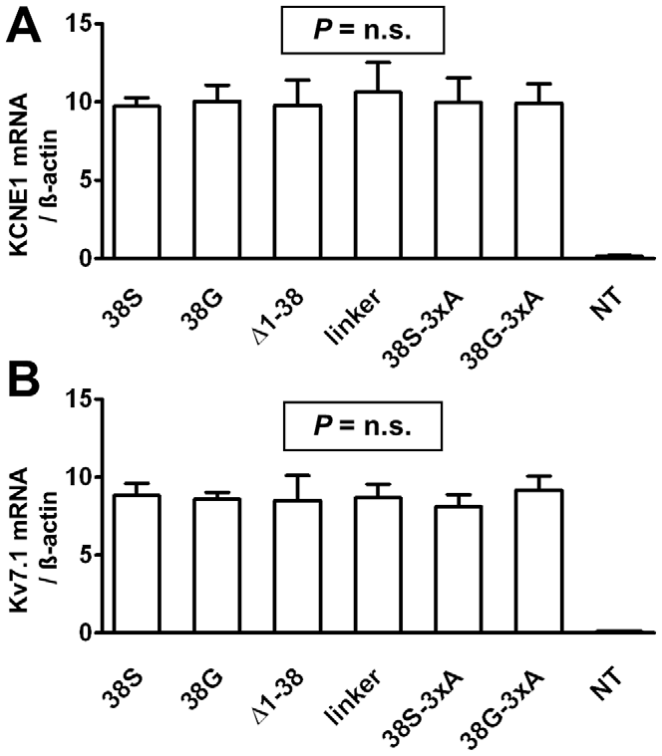
mRNA expression levels. Mean±SEM results of quantitative real-time PCR for mRNA expression of KCNE1 (**A**) and Kv7.1 (**B**) from HEK cells transfected with Kv7.1 and the various KCNE1 constructs (n = 5 transfections for each panel). Data were normalized to ß-actin expression. There were no differences in mRNA transcription that could account for changes in membrane currents.

We excluded variation in levels of transcription in this heterologous system as a confounding factor. All KCNE1 constructs showed similar mRNA levels (‘38S’: 9.7±0.6, ‘38G’: 10.0±1.2, ‘Δ1-38’: 9.8±1.9, ‘linker’: 10.6±2.2, ‘38S-3xA’: 10.0±1.8, ‘38G-3xA’: 9.9±1.4; non-transfected control: 0.2±0.1 AU; *P* = n.s.; Fig. 7). Co-transfection of KCNE1 constructs did not change Kv7.1 mRNA expression (‘38S’: 8.8±1.0, ‘38G’: 8.6±0.5, ‘Δ1-38’: 8.5±1.9, ‘linker’: 8.7±1.1, ‘38S-3xA’: 8.1±1.0, ‘38G-3xA’: 9.2±1.1; non-transfected control: 0.1±0.0 AU; *P* = n.s.; Fig. 7).

## Discussion

### Major findings

This study identifies the importance of three arginines in the N-terminus of KCNE1 (positions 32, 33 and 36) for plasma-membrane localization of the I_Ks_ channel complex. These residues contribute to membrane representation of the β-subunit together with the α-subunit. Removal of these arginines by alanine-scanning in KCNE1-38S led to a comparable I_Ks_ phenotype as observed with the atrial fibrillation-related KCNE1-38G or the construct with N-terminal KCNE1 truncation – suggesting a link between a sterical KCNE1-38G alteration mediated by glycine (increasing protein mobility by formation of a glycine hinge) and a failure of N-terminal arginines to get in contact with the membrane. Structural disruption at position 38 by introducing a ‘linker’ might have similarly affected the ability of arginines to interact with the plasma-membrane.

### Relation to delayed rectifier channel trafficking

Kv7.1 and KCNE1 proteins are highly expressed in human atria and ventricles and the resultant I_Ks_ is physiologically relevant for cardiac repolarization [4]. The results of our study illustrate that plasma-membrane localization of the α-subunit Kv7.1 and the ß-subunit KCNE1 was dependent on the presence of positively-charged arginines within the KCNE1 N-terminus. Trafficking of the α-subunit Kv7.1 can be regulated differently when co-assembled with KCNE1. Krumerman et al. found a mutation (KCNE1-L51H) in the transmembrane domain of the β-subunit that conferred trafficking deficiency. KCNE1-L51H did not leave the endoplasmic reticulum and resultant I_Ks_ was diminished [13]. In line with this, subsequent work suggested KCNE1 as a chaperone for Kv7.1 intracellular trafficking by protecting the α-subunit from proteasomal degradation [14]. Conversely, Chandrasekhar *et al*. documented evidence for the necessity of Kv7.1 for KCNE1 membrane expression arguing that Kv7.1 may in fact be needed to help KCNE1 to the membrane [15].

Our results are consistent with previous work that indicated reduced plasma-membrane localization for the atrial fibrillation-associated ‘38G’ allele reducing resultant I_Ks_ [12]. In the present study we identified three arginines at positions 32, 33 and 36 that are important for plasma-membrane representation of the I_Ks_ complex. Positively-charged amino-acids may interact with hydrophilic phospholipid components of cellular membranes [16]. Arginine is typically located at membrane-water boundaries [17]. Several studies have identified a respective role for arginine in membrane localization. For instance, Jaskolski *et al.* described splice variants of a glutamate receptor where the variant containing a sequence of two arginines and two lysines showed significantly higher membrane localization [18]. Furthermore, membrane anchoring of saposin-C depends heavily on two lysines of the protein. Substitution of either lysine with neutral or negatively-charged amino-acids led to decreased anchoring while exchange with arginine intensified membrane anchoring [19]. Consistently, substitution of the three N-terminal KCNE1 arginines resulted in reduced current density and plasma-membrane localization in the present work. The reduction of membrane localization was comparable to N-terminal truncation of KCNE1 and glycine substitution suggesting a role of these amino-acids for KCNE1 function.

Furthermore, substitution of serine at position 38 with glycine reduced I_Ks_ in a similar fashion as deletion of amino-acids 1-38 – the greater part of the N-terminus. Results were similar when residue 38 was substituted by a series of five alanines. This construct was synthesized in order to mimic the effect of increased mobility by steric distortion of the protein. Glycine is smaller than serine and does not enable covalent binding [20]. It might therefore act as a hinge rendering the N-terminus more flexible, potentially leading to reduced membrane anchoring due to steric alterations of this KCNE1 region.

### Effect of KCNE1 on I_Ks_ properties

The KCNE1 transmembrane domain interacts with the pore region [6] and with the transmembrane segment 6 [7], [21], [22] of the α-subunit Kv7.1.

Amino-acids 57–59 of KCNE1 importantly modulate I_Ks_ activation kinetics [23]. In subsequent work these investigators found that a hydroxylated central amino acid (threonine 58) was necessary for the slow sigmoidal activation produced by KCNE1 together with Kv7.1. An aliphatic amino acid substituted at position 58 of KCNE1 led to a pronounced change in activation properties [7]. The more rapid activation of I_Ks_ resulting from co-transfection of Kv7.1 and 38G in this paper cannot be explained by these sterical alterations.

Recent work indicated an interaction of KCNE1 with the Kv7.1 voltage sensor [24], [25]. Furthermore, I_Ks_ is very sensitive to mutations in the KCNE1 C-terminus [26], [27] and several of these mutations have been linked to the long QT syndrome [28]. The C-terminus of KCNE1 interacts directly with the pore region of Kv7.1 and modulates I_Ks_ kinetics and current amplitude [29]. Studies using disulfide bonding also indicated potential interaction of Kv7.1 transmembrane segment 1 with the KCNE1 N-terminus (amino-acids 40, 41) [30].

Two of the KCNE1 constructs studied in the present paper (‘linker’ and ‘38S-3xA’), slowed channel deactivation. A study by Melman et al. located a sequence in the KCNE1 transmembrane domain (amino-acids 48–56) that was important for the deactivation process of I_Ks_ [23]. Changes in the KCNE1 N-terminus on the other hand reduced I_Ks_, but had no effects on kinetics. Kinetic changes observed in our study were only evoked by two of the constructs and not by the N-terminally truncated protein. Slowed deactivation in these cases might have been due to indirect conformational changes potentially influencing the aforementioned transmembrane sequence but further studies are needed to understand this phenomenon.

### Relevance for atrial fibrillation

Chen et al. reported a gain-of-function mutation in Kv7.1 (S140G) that was associated with increased atrial fibrillation prevalence among members of a large Chinese family [31]. In fact, this was the first description of a monogenetically inherited form of atrial fibrillation. While I_Ks_ gain-of-function may abbreviate action potential duration and refractoriness and therefore represent a pro-arrhythmic substrate, loss-of-function is less well intuitively correlated with the occurrence of atrial fibrillation. In clinical studies, the KCNE1 single nucleotide polymorphism G38S has been associated with atrial fibrillation [10]. Previous modelling studies of I_Ks_ with KCNE1 polymorphism alleles showed the generation of early after-depolarisations with the atrial fibrillation-associated ‘38G’ under specific conditions that might cause enhanced automaticity and thus trigger the arrhythmia [12].

Clinical observations provide consistent evidence for a physiological consequence of the single nucleotide polymorphism as fibrillatory rates in atrial fibrillation-patients were slower for those with the ‘38G’ allele in comparison to patients with the ‘38S’ allele [32]. Slower fibrillatory rates relate to prolonged refractory periods may well be associated with reduced repolarizing currents.

### Limitations

The conclusion of the present study relies on indirect evidence that positively charged amino-acids may need to be in contact with the membrane for increasing membrane representation of the I_Ks_ delayed rectifier channel complex. Direct evidence from crystallography would be highly desirable but is currently unavailable. Further, with the present study we cannot exclude slower degradation of ion channel complexes with the KCNE1-38S isoform underlying increases in I_Ks_ and enhanced channel membrane representation.

### Conclusion

We have performed a detailed electrophysiological and biochemical analysis of structural determinants underlying reduced I_Ks_ with the atrial fibrillation-related single nucleotide polymorphism KCNE1-38G. The results of this study suggest that the KCNE1 N-terminus is engaged in I_Ks_ ion channel complex plasma-membrane representation. N-terminal positively-charged arginine residues 32, 33 and 36 play a specific and important role in the reconstitution of I_Ks_. Presence of glycine at position 38 and substitution of arginines mimic properties (reduced current density and plasma-membrane localization) as seen with N-terminal truncation of KCNE1 arguing for the specific importance of these arginines for physiological I_Ks_ function.

## Methods

### Site-directed mutagenesis

KCNE1-38G (‘38G’, GenBank NM_000219) was flanked by restriction sites HindIII and EcoRI by polymerase chain reaction (PCR) using primers shown in Table 1. These primers were used to introduce restriction sites into all constructs (Fig. 1B). The KCNE1 polymorphism ‘38S’ was already subcloned into pcDNA3.1 [12]. Standard cloning technique (Table 1) was used. Additionally, each construct was flag-tagged C-terminally. All constructs were checked by sequencing.

**Table 1:** Primers for KCNE1 mutagenesis.

Primer	Constructs	Comments	Sequence
Fw1	all constructs	introduction of restriction site	5′-CGCAAGCTTATGATCCTGTC-3′
Fw2	Δ1-38	truncation of AA 1-38	5′-*CGCAAGCTTATG*GACGGCAAGCTGGAG-3′
Fw3	linker	overlap extension	5′-*CAGCAGCAGCAGCA*GACGGCAAGCTGGAG-3′
Fw4	Intermediate construct 38S-R36A	overlap extension	5′-CAGGTCCCC*AGC*CAGCAGTGACGGCAAG-3′
Fw5	Intermediate construct 38G-R36A	overlap extension	5′-CAGGTCCCC*AGC*CAGCGGTGACGGCAAG-3′
Fw6	38S-3xA	overlap extension	5′-GCCTGGCC*GCAGC*GTCCCCAGCCAGCAGTG-3′
Fw7	38G-3xA	overlap extension	5′-GCCTGGCC*GCAGC*GTCCCCAGCCAGCGGTG-3′
Rev1	all constructs	Introduction of restriction site	5′-CTCGAATTCTCATGGGGAAGGC-3′
Rev2	linker	overlap extension	5′-*TGCTGCTGCTGCTG*CGCTGCGGGGGGACCTGC-3′
Rev3	Intermediate construct 38S-R36A	overlap extension	5′-CACTGCTG*GCT*GGGGACCTG-3′
Rev4	Intermediate construct 38G-R36A	overlap extension	5′-CACCGCTG*GCT*GGGGACCTG-3′
Rev5	38S-3xA, 38G-3xA	overlap extension	5′-GGGAC*GCTGC*GGCCAGGCCCGACAT-3′
Rev6	flag-tagged constructs	C-terminal flag-tag	5′-*CTCGAATTCTCACTTGTCGTCGTCGTC CTTGTAGTC*TGGGGAAGGCTTCGTC-3′

Mutation containing sequences in italic, underlined.

Both naturally occurring alleles of the polymorphism (‘38S’ and ‘38G’) had previously been cloned [12]. Alanine-substitution of the three N-terminal KCNE1 arginines was performed to disrupt these positively charged amino-acids that might play a role in membrane anchoring (‘38S-3xA’, ‘38G-3xA’). N-terminal truncation of KCNE1 was performed to study the role of the greater part of the N-terminus (‘Δ1-38’). Glycine substitution at position 38 by four alanines (‘linker’) was performed in order to mimic the effect of increased mobility by steric distortion of the β-subunit. Glycine might act as a hinge to allow flexibility of the N-terminus, potentially leading to reduced membrane anchoring due to steric alterations of this KCNE1 region.

### Cell culture

Human embryonic kidney (HEK) and Chinese hamster ovary (CHO) cells (ATCC, Manassas, VA, USA) were cultured at 37°C with 5% CO_2_ in MEM or F12-medium (Invitrogen, Carlsbad, CA, USA) supplemented with 10% heat-inactivated fetal bovine serum. Transient transfections were obtained with polyethylenimine (Polysciences Inc., Warrington, PA, USA) and a total of 1 µg cDNA for electrophysiological measurements, 0.5 µg for protein-biochemistry and 0.25 µg cDNA for confocal microscopy. Co-transfected green fluorescent protein (0.2 µg cDNA) served as a transfection marker for electrophysiological recordings. All experiments were performed 48 h after transfection.

### Electrophysiological recordings

Currents were recorded with whole-cell patch-clamp at physiological temperature (36.5±0.5°C) with an Axopatch 200B amplifier and pClamp9.1 software (Molecular Devices, Sunnyvale, CA, USA). Borosilicate glass electrodes had 1.5 to 3.0 MΩ tip resistances when filled with internal solution. Compensated cell capacitances were 27.0±2.1 pF with no statistical differences between cells transfected with respective KCNE1 constructs. Liquid junction potentials (7.5±0.5 mV) were not corrected. The extracellular solution was composed of (mM) NaCl 136, KCl 5.4, MgCl_2_ 1, CaCl_2_ 1, NaH_2_PO_4_ 0.33, HEPES 5 and dextrose 10 (pH 7.35 with NaOH). Internal solution contained (mM) K-aspartate 110, KCl 20, MgCl_2_ 1, MgATP 5, GTP (lithium salt) 0.1, HEPES 10, Na-phosphocreatine 5 and EGTA 5.0 (pH 7.3 with KOH). Currents were elicited with 1 s depolarizing pulses from a holding potential of −80 mV and an inter-pulse interval of 12 s. Tail currents were observed during 2 s repolarizations to −50 mV from various depolarizing potentials.

Clampfit (Molecular Devices, Sunnyvale, CA, USA) and GraphPad Prism (GraphPad Software, San Diego, CA, USA) were used for data analysis. Data are presented as mean±SEM. *P*<0.05 by two-way ANOVA (for comparison among groups) or Student's *t*-test (for comparison of individual voltage steps) was considered to indicate statistical significance. We assumed Student's *t*-test to be sufficiently precise even in the case of unequal variance as sample sizes were similar among different groups.

### Protein biochemistry

HEK cells were washed with ice-cold phosphate-buffered saline (pH 8.0) to remove media and scraped into lysis-buffer composed of (mM) Tris-HCl (pH 7.4) 5, EDTA 2, and phenylmethanesulfonyl fluoride 1 and protease inhibitors (mg/ml): benzamidine 10, leupeptine 5, trypsin inhibitor 5. Cells/proteins were continuously kept at 4°C. After homogenization (PowerGen125, Fisher Scientific, Waltham, MA, USA) for 2×10 s cells were centrifuged at 1,000 *g* for 5 min. The resulting supernatant contained crude membranes and was centrifuged at 28,000 *g* (20 min). The pellet was resuspended in lysis-buffer, washed, suspended in Laemmli buffer, and loaded onto sodium dodecyl-sulphate (SDS) gels. Proteins were transferred to nitrocellulose membranes and stained with primary antibodies (anti-flag, anti-spectrin, anti-actin Sigma Aldrich, St. Louis, MO, USA; anti-Kv7.1, Alomone, Jerusalem, Israel; anti-calnexin, Abcam, Cambridge, MA, USA; all 1∶1,000). After secondary antibody incubation (anti-goat and anti-rabbit, EMD4 Biosciences, Gibbstown, NJ, USA; anti-mouse, Santa Cruz Biotechnology, Santa Cruz, CA, USA) bands were visualized with chemiluminescence.

Cells were treated with the ‘Membrane Protein Extraction Kit’ (Promokine, Heidelberg, Germany) to obtain plasma-membrane and endoplasmic reticulum fractions. Equal loading and accuracy of fractionation was probed by immunodetection of calnexin as endoplasmic reticulum marker and spectrin for plasma-membranes. Protein expression was normalized to calnexin or spectrin.

### Immunoprecipitation

Transfected HEK cells were washed with phosphate-buffered saline and scraped into 1 ml buffer containing (mM) Tris-HCl (pH 7.4) 50, NaCl 150, EDTA 1, EGTA 1, PMSF 1 and 1% NP-40, 0.5% sodium deoxycholate, 0.1% SDS as well as protease inhibitors as above. Again all steps were at 4°C. After 1 h incubation under rotation the cells were centrifuged for 10 min at 2,800 *g*. Samples were precleared with 25 µl protein-G-agarose beads (Pierce Biotechnologies, Rockford, IL, USA) for 30 min under rotation. Beads were removed by 1 min centrifugation at 1,000 *g*. 1 µg of antibody was added to 2 mg of supernatant and incubated overnight. Another 30 µl protein-G-agarose beads were added and incubated for 3 h. The beads were sedimented by 1 min centrifugation at 1,000 *g*, washed and suspended in Laemmli buffer before loading onto SDS gels.

### Confocal microscopy

Transfected HEK cells were grown on plastic coverslips (ibidi, Martinsried, Germany) and fixed (20 min) with 2% paraformaldehyde (Sigma-Aldrich, St. Louis, MO, USA). After 3 washes (5 min) with phosphate-buffered saline cells were blocked with 5% normal donkey serum (Jackson ImmunoResearch Laboratories, West Grove, PA, USA) and 5% bovine serum albumin (BSA, Sigma-Aldrich, St. Louis, MO, USA). Then cells were permeabilized with 0.2% Triton X-100 (Sigma-Aldrich, St. Louis, MO, USA) for 1 h. Primary antibodies were incubated overnight at 4°C: rabbit anti-flag (Sigma Aldrich, St. Louis, MO, USA), goat anti-Kv7.1 (Santa Cruz Biotechnology, Santa Cruz, CA, USA) and mouse anti-pan-cadherin (Abcam, Cambridge, UK) at 1:200 dilution, followed by 3×5 min washes and a 1 h incubation with secondary antibodies (fluorescence-labelled ‘Alexa-Fluor-555’ [anti-rabbit] for KCNE1 and ‘Alexa-Fluor-488’ [anti-goat] for Kv7.1). Confocal microscopy was performed with a Zeiss LSM-510 system. KCNE1 (green) and Kv7.1 (red) were excited at 555 and 495 nm with Helium/Neon- and Argon-Lasers, respectively, emitting fluorescence at 565 and 519 nm. Confocal microscopy experiments of different groups were performed on the same experimental day with identical parameters for all groups. Control experiments omitting primary antibodies and with non-transfected cells revealed absent or very low-level background staining.

### RNA extraction, reverse transcription and RNA quantitation

RNA was extracted from transfected HEK cells using the Roti-Quick-Kit (Roth, Karlsruhe, Germany). cDNA was synthesized from 1 µg of total RNA. Reactions (20 µl) contained 1.25 µM random hexamers, 1.25 µM oligo-dTs, 0.25 mM each of dNTPs, 200 U reverse transcriptase (Invitrogen, Carlsbad, CA, USA), reverse transcriptase buffer, 10 mM dithiothreitol (DTT) and 40 U RNAse inhibitor. RNA, random hexamers, oligo-dTs, dNTPs, and RNAse inhibitor were incubated (65°C, 5 min), then put on ice (5 min). Reverse transcriptase buffer and DTT were added and incubated (37°C, 2 min). This was followed by 50 min incubation at 37°C and then 15 min incubation at 70°C. Real-time PCR was performed using the 7300 Fast Real-Time PCR System and the Fast SybrGreen Master Kit (Applied Biosystems, Foster City, CA, USA). Reactions (20 µl) contained cDNA (1 µl) and 0.5 µM of each forward and reverse primer (Table 2). A “20 s denaturation” at 95°C was followed by 40 cycles of denaturation (1 s) at 95°C and annealing and elongation (20 s) at 60°C.

**Table 2:** Primers for quantitation of Kv7.1 and KCNE1 mRNA.

Primer	GenBank accession no.	Sequence
Kv7.1	NM_000218	F: 5′-AGACGTGGGTCGGGAAGAC-3′R: 5′-CAAAGAAGGAGATGGCAAAGACA-3′
KCNE1	NM_000219	F: 5′-GAGGCCCTCTACGTCCTCATG-3′R: 5′-TGATGCCCAGGGTGAAGAA-3′
ß-actin	NM_001101	F: 5′-TGGATCAGCAAGCAGGAGTATG-3′R: 5′-GCATTTGCGGTGGACGAT-3′

F – forward primer, R – reverse primer.
